# Fractal construction of constrained code words for DNA storage systems

**DOI:** 10.1093/nar/gkab1209

**Published:** 2021-12-15

**Authors:** Hannah F Löchel, Marius Welzel, Georges Hattab, Anne-Christin Hauschild, Dominik Heider

**Affiliations:** Department of Mathematics and Computer Science, University of Marburg, Germany; Department of Mathematics and Computer Science, University of Marburg, Germany; Department of Mathematics and Computer Science, University of Marburg, Germany; Department of Mathematics and Computer Science, University of Marburg, Germany; Department of Mathematics and Computer Science, University of Marburg, Germany

## Abstract

The use of complex biological molecules to solve computational problems is an emerging field at the interface between biology and computer science. There are two main categories in which biological molecules, especially DNA, are investigated as alternatives to silicon-based computer technologies. One is to use DNA as a storage medium, and the other is to use DNA for computing. Both strategies come with certain constraints. In the current study, we present a novel approach derived from chaos game representation for DNA to generate DNA code words that fulfill user-defined constraints, namely GC content, homopolymers, and undesired motifs, and thus, can be used to build codes for reliable DNA storage systems.

## Graphical Abstract

**Graphical Abstract:**
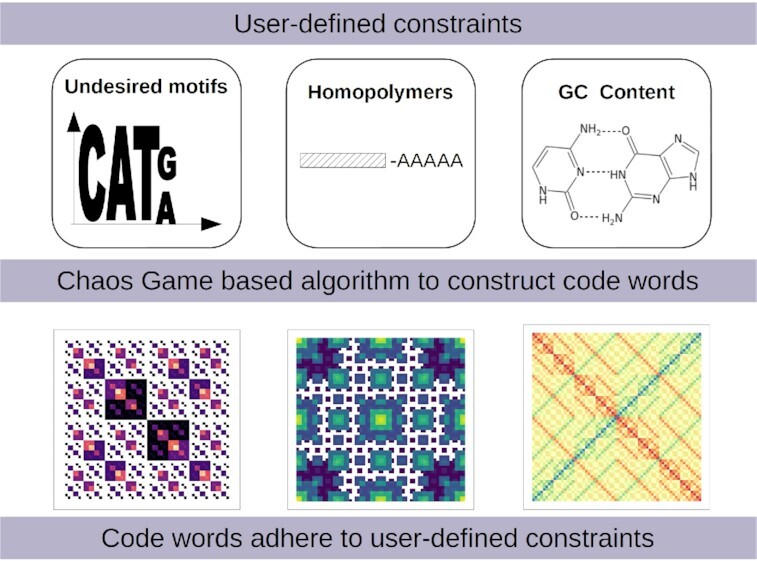
Fractal construction of constrained code words for DNA storage systems.

## INTRODUCTION

Due to the increasing speed of global digitization, the amount of digital data produced is growing exponentially (1). Conventional storage mediums such as hard disks have a maximum information density of about 10^3^GB/mm^3^ (2). However, their life expectancy is rather short, and thus, only magnetic tapes are used for long-term storage. Since these tapes also have a very short life expectancy, they are copied every five years on average to guarantee data safety. An alternative data storage medium exists in nature in the form of deoxyribonucleic acid (DNA), which consists of the four nucleotides adenine (A), thymine (T), guanine (G) and cytosine (C). DNA has an estimated information density of about 4.6 × 10^8^GB/mm^3^ and is, under optimal conditions, stable for thousands of years (2). Several groups have developed approaches for DNA data storage (1,3–9) and DNA watermarking (10–15). However, limitations in code word design have only been partly addressed so far.

To store information into DNA, the binary information is encoded into DNA sequences (code words) in the first step. In the next step, these sequences are synthesized and stored. The DNA can be sequenced at any time to retrieve the stored information (16). The development of Next-Generation Sequencing (NGS) technologies allows a quick reading of the information, while the DNA synthesis remains a limitation for DNA data storage, as the synthesis is very time and cost consuming. Thus, DNA storage systems are not competitive for commercial use at the moment. However, it is expected that the costs for synthesis will drop significantly in the near future (17). Nevertheless, DNA storage systems allow easy and low cost copying of media, in contrast to conventional storage systems. A copy of a conventional storage medium efforts the same price as the original medium. For DNA, the first synthesis is relatively expensive, while copies can be achieved at a very low price. The goal of this paper is to describe a novel approach for the construction of codebooks (set of code words), adhering to user-defined constraints, which can be incorporated into any existing error-correction algorithms. To this end, the code word library design can be done separately from the subsequent error-correction codes in some cases, e.g. for the proposed method, which is an advantage regarding computational complexity compared to other codes where unwanted motifs need to be discarded during encoding. In our approach, the code word design is independent of the subsequent error-correcting codes. Thus, we do not address error correction explicitly in our analysis.

DNA synthesis and sequencing methods are error-prone (18), in particular, if the DNA sequence contains specific patterns. Common synthesis approaches first synthesize short fragments (oligos) of the target sequence, which are subsequently assembled. The assembly process requires that the oligos have a similar GC content and, if homopolymers exist, that they are few in number and rather short (19). Sequencing methods are another potential error source (20), with error probabilities increasing for sequences containing long stretches of homopolymers, a strongly deviating GC content, or method-dependent motifs (19).

Thus, due to limitations in the DNA synthesis and sequencing processes, the nucleotide composition of synthetic DNA fragments that can be used for data storage is subject to multiple constraints to reduce errors in, e.g. DNA synthesis and DNA sequencing.

These constraints depend on the chosen methodologies for synthesis, sequencing, and also storage but can be roughly divided into restrictions regarding the GC content of the sequences, homopolymers (hp) and undesired motifs (19).

- **GC content** must either be constant (strongly constrained) or within a certain interval (weakly constrained) (21) to reduce the probability of secondary structure formation and to ensure uniform sequence coverage in the sequencing (22).
- **Homopolymers** are continuous repeats of a certain nucleotide that can lead to increased error rates in sequencing methods (23), as sequencing methods often fail to recognize the correct lengths of homopolymers. Thus, the length of homopolymers should be limited for DNA code words in DNA storage systems.
- **Motifs** are short subsequences in a DNA sequence. Sequencing and synthesis methods depend on short DNA motifs to initiate the amplification steps of the workflows (24,25). Due to these limitations in synthesis, respective motifs have to be excluded (e.g. restriction sites (26)). Moreover, other motifs can introduce errors throughout the sequencing process, for instance, due to secondary structure formation (19).

Due to the fact that DNA-based storage systems are in their early stages of development, further constraints may arise based on the experimental conditions, such as new sets of motifs for novel synthesis or sequencing technologies, as well as for novel concepts such as *in vivo* DNA storages.

To take these limitations and constraints into account, a flexible code word design is required for DNA storage systems. Various deterministic approaches adhering to the homopolymer and GC content constraints exist, for instance in (21,27–30). Other heuristic methods, e.g. in (16,31–34), additionally take into account a large minimal Hamming distance (the number of positions that differ between two strings).

However, one important constraint that is overlooked in the existing literature is that of undesired motifs, which are important for the synthesis, sequencing, and storage of DNA sequences. This is particularly relevant since the amount and composition of these motifs largely depend on the employed DNA manipulation techniques, for instance, primer targets for random access or certain motifs with biological relevance for *in vivo* storage (1,2). Accordingly, solutions for codebook design are required that allow the flexible creation of code words while respecting various motif constraints. This enables the evaluation of different combinations of synthesis, sequencing, and storage techniques.

In our study, we developed a fractal-based method to generate all possible code words that does not only take into account GC content and homopolymer constraints, but also the challenge of excluding user-specified motifs. Our method is based on a modified frequency matrix chaos game representation (FCGR), an extension of the chaos game representation (CGR), which transforms a DNA sequence into a fractal.

The term fractal was introduced by Mandelbrot to describe self-similar geometrical forms (35). Moreover, he described fractals as a set for which the Hausdorff dimension exceeds the topological space. For a self-similar geometrical form, the structural patterns of the form can be found in small sections of the form in a repetitive manner. In ideal fractals (in a mathematical sense), this self-similarity is infinite (36).

**Figure 1.**
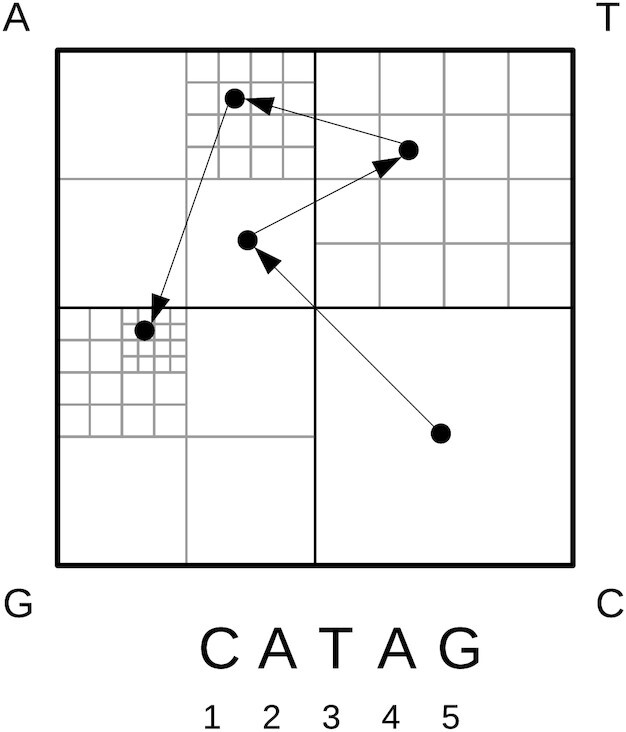
Chaos game representation. Example mapping for creating a CGR based on a given input sequence: *CATAG*.

The chaos game representation (CGR) was first described by Barnsley (37). It is an iterative function system to construct fractals. The underlying idea of the algorithm is to assign numbers from one to three to the edges of a triangle. The algorithm starts from a randomly chosen vertex. Then the next vertex is drawn by randomly choosing a number from one to three, representing an edge of the triangle. Half of the distance to the direction (so-called scaling factor) to this edge of the triangle is drawn to set the new vertex. After several iteration steps, the Sierpinski triangle appears. Abbreviations of the algorithm, such as a change of angle, the scaling factor, or the number of edges, result in differently shaped fractals (37). The resulting patterns are called chaos game representation (CGR). Jeffrey (38) was the first who used the CGR algorithm for DNA, where the four nucleotides are assigned to the edges of a square (see Figure 1). There are several applications based on CGR (39), e.g. the analysis and comparison of whole-genome sequences (40) or phylogenetic predictions (41). Moreover, the CGR has some interesting properties, as pointed out by Almeida et al. (42). The CGR patterns are unique, the sequence can be reconstructed by the coordinates, and the distributions of the points can be described as a generalized Markov Chain model.

**Figure 2.**
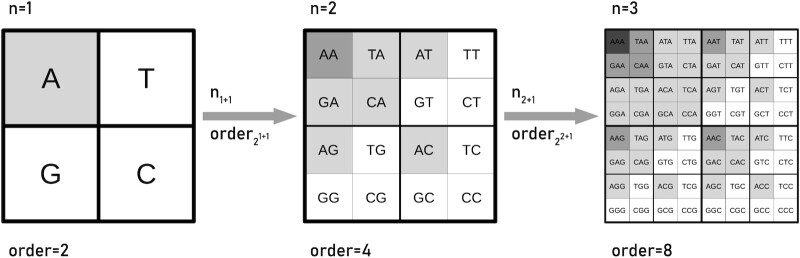
Chaos game representation matrices for increasing word length (n). With the extension of CGR to FCGR in a matrix order of 2^*n*^ (mCGR), each element in the matrix represents a word/string of the given length. The increasing order can be achieved by Kronecker powers. The single characters of the strings have a fractal order. The example is shown for the fractal pattern of the count of the nucleotide *A*.

The frequency matrix chaos game representation (FCGR) is an extension (42) of the CGR, in which the CGR is represented as a count matrix, where the number of dots of the CGR in each section of a grid is counted. The FCGR is particularly useful for machine learning approaches as it provides a fixed input dimension for any sequence length and has been successfully applied already in protein classification (43). An FCGR with the order of 2^*n*^ represents a matrix of *k*-mers. Based on that, we can consider it as a representation of all possible DNA words of the length *n* (see Figure 2). We will refer to this matrix representation in the order of 2^*n*^ as matrix chaos game representation (mCGR) in the following. As shown in Figure 2, the number of entries in the matrix increases by 2^*n*^ with increasing order. The coloring of the nucleotide *A* shows an emerging fractal pattern. The white elements in the matrix build the Sierpinski triangle. The different gray values represent the amount of *A* in a sequence with a self-similar pattern. The simple case of a word length *n* = 1 is a representation of the four nucleotides $\overset{A}{G}\ \overset{T}{C}$. The increasing word length can be represented as Kronecker power (Kronecker Product of the matrix by itself) with the following definition:

(1) $$\begin{equation*} M^n=M \otimes M \otimes M ... \end{equation*}$$

In CGR (or mCGR, respectively), the letter is appended to calculate the strings in CGR (or mCGR). For prepending the characters to the string, the representation is also known as genomatrices (44–46). Both notations, CGR/mCGR as well as genomatrices, have been used to demonstrate that fractal patterns emerge from motifs (47,48). Since the positional information of the matrix elements can be used to reconstruct the underlying sequence, the matrix elements can carry additional information, e.g. whether the use of the corresponding sequence is restricted. Anitas (48) assigned probabilities to the squares and applied the Kronecker powers to perform structural analyses of DNA by CGR. While the Kronecker power results in a multiplication of the probabilities, we propose an addition of the substrings instead of a multiplication of probabilities.

Based on this initial idea, we developed and optimized a model to generate a set of all possible code words of a given length which adhere to various constraints, such as limitation of homopolymers, undesired motifs, and variable GC content constraints (both weakly, i.e. in an interval, and strongly, i.e. as a fixed percentage), and thus can be used and integrated into codes for DNA storage systems. To this end, we compared our approach with existing methods for code word design, namely (16,27–31,33,34). Moreover, we integrated our codebooks into a lexicographic encoding (which maps a binary sequence to DNA (29)) as a proof-of-concept, and compared its performance with current state-of-the-art algorithms, namely DNA Fountain codes (3). However, our CGR-based codebook design can be used as a basis for any DNA storage codes, e.g. error-correction codes. We provide a Java implementation and R scripts available at http://mCGR.heiderlab.de and source code at https://github.com/HFLoechel/ConstrainedKaos.

The Hamming distance, which is particularly important for error-correction and DNA-computing, a field that is closely related to DNA storage systems, can also be easily calculated with our model.

## MATERIALS AND METHODS

Our approach is based on CGR, and we used the concept of CGR to implement a mathematical model for designing code words that fulfill certain constraints that are important for DNA storage systems, namely homopolymers, undesired motifs, and GC content, as well as additional constraints that are necessary for DNA computing, namely the Hamming distance of code words.

### Homopolymers and undesired motifs

Homopolymers are defined as continuous repeats of a certain nucleotide and may lead to increased error rates in sequencing methods (23). Thus, it is important that homopolymers are excluded from code words for DNA data storage systems.

**Figure 3.**
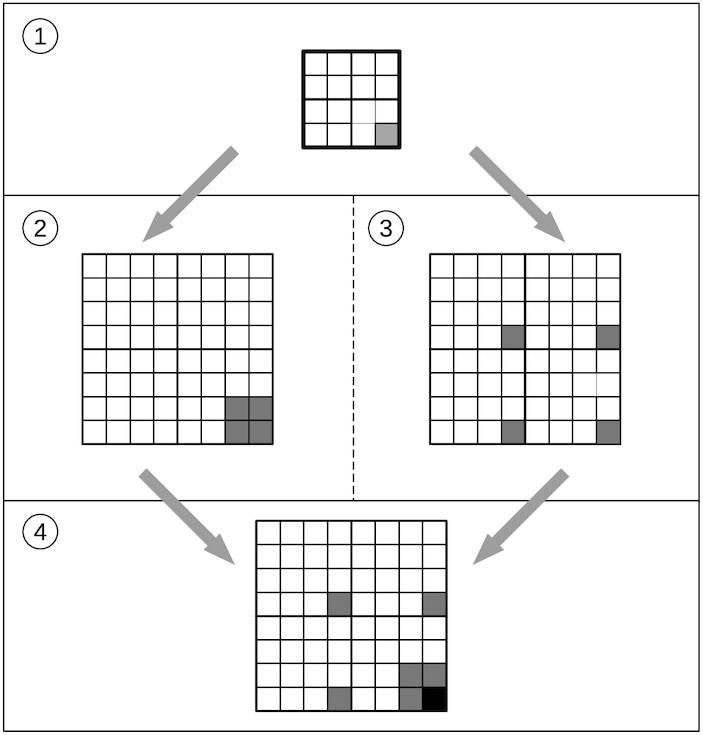
Algorithm for constrained subsequences. (1) mCGR with the constraining motif (here *CC*) is initialized. The matrix size is given by the length of the constraint. (2) The mCGR is scaled to double the size and (3) used for tiling. (4) The result is added and can be used for the next iteration until the order of the matrix for the desired code word length is reached.

In each quarter of an mCGR, every sequence ends with the same letter, for any order of the matrix. Moreover, mCGRs as fractals are self-repetitive. Consequently, a repetition of a particular pattern for any given subsequence exists. An example demonstrating the construction of an mCGR for the homopolymer *CC* is shown in Figure 3. This approach cannot only be used for the exclusion of homopolymers but also for any undesired motif.

Thus, for any given sequence constraint, we first create an mCGR of the last coordinate in the order of the constrained length, where we denote the element of the excluded sequence with 1 and the remaining elements with 0. This initial matrix serves as an input or generator matrix for the next iteration step. The generator matrix is first used for tiling over a matrix for the next word length. In the second step, the generator matrix is stretched to the order for the next word length and added to the matrix of the first step. This results in the next generator matrix for the next iteration step. This procedure will be repeated until the desired sequence length is reached. For more than one constraint, this procedure can be repeated and the resulting matrices can be added. In Figure 3, an example for homopolymer *CC* is shown, wherein step 1 the last matrix element is denoted with one. A homopolymer with the same length can be added by matrix addition. For instance, for all homopolymers with a sequence length of two, the corresponding generator matrix is 
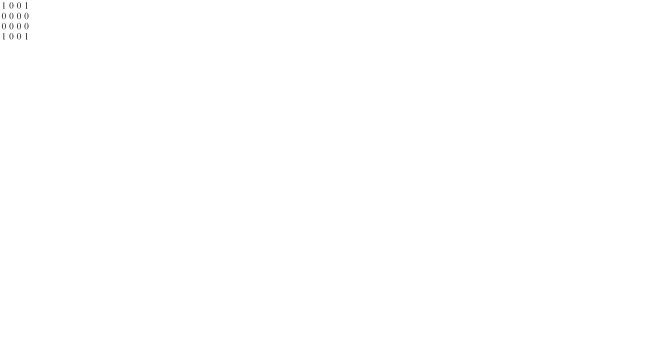
. For the addition of a constraint with a length of 3, the mCGR of an order 2^3^ has to be calculated, and both matrices have to be combined by matrix addition.

Thus, matrices with increasing orders of 2^*n*^ are representations of all sequences of a given word length *n*. But instead of using a matrix of characters, we use a representation of ones and zeros for the generator matrix *mCGR*^*n*^ (where *n* indicates the length of the initial word).

In contrast to Equation (13), where we append a string with a single character, we have to take the complete mCGR of the initial iteration step into account (see Figure 3). Thus, we have to exchange the appending part of the equation (Equation (12)). We can achieve this by mirroring the prepending part (Equation (11)):

(2) $$\begin{equation*} mCGR^n= {1\!\!1}^{2^1} \otimes mCGR^{n-1}+mCGR ^{n-1} \otimes {1\!\!1}^{2^1} \end{equation*}$$

where ${1\!\!1}^n$ represents the square matrix of ones with an order of *n*. The matrix elements that are not affected by the constraints remain zero, and the remaining elements are counting the numbers of the events where constraints appear.

### GC content

**Figure 4.**
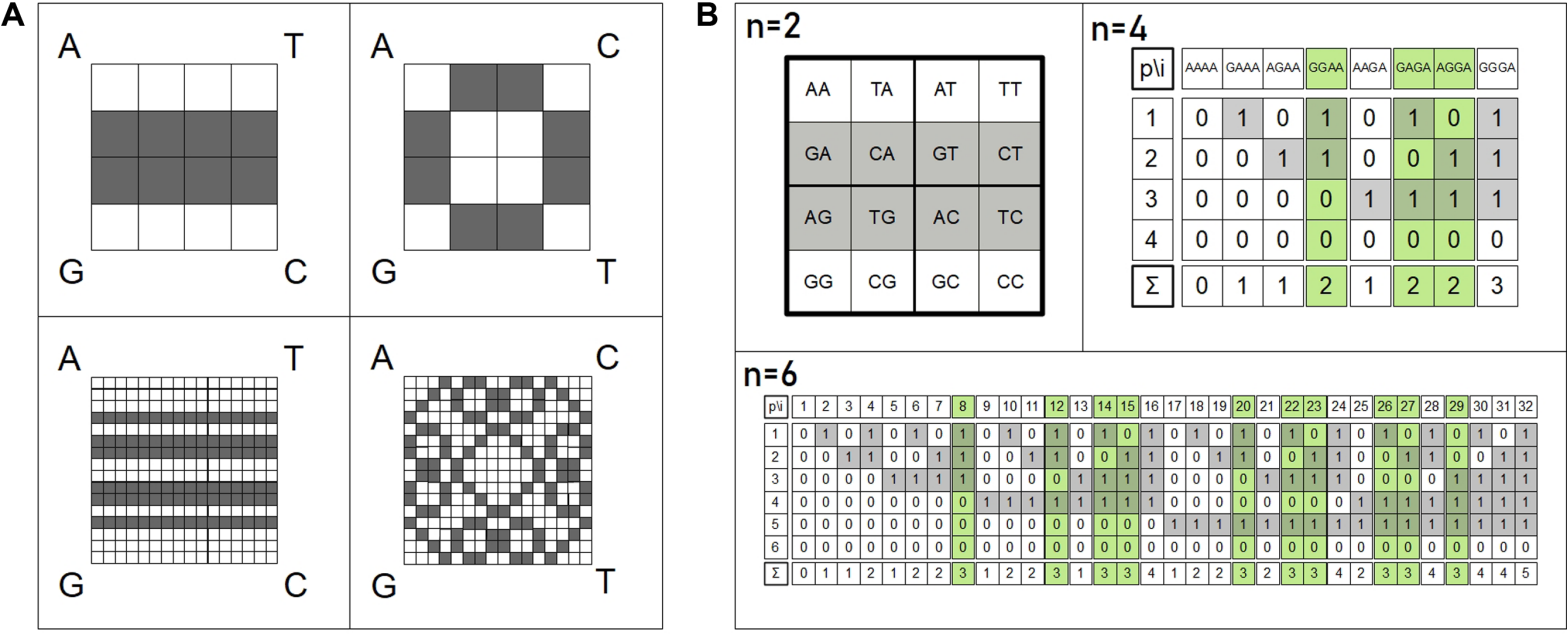
GC content. (**A**) Patterns for 50 % GC content for the word length *n* = 2 (top) and *n* = 4 (bottom). The resulting matrices vary, depending on the order of nucleotides at the edges. (**B**) Calculation of the GC content, shown for word length *n* = 2, *n* = 4, and *n* = 6. For *A* = *T* = 0, *G* = *C* = 1, and an order of $\overset{A}{G}\ \overset{T}{C}$, bars emerge by coloring 50% GC content. The sum of the characters in a string can be used to calculate the amount of *A* = *T* and *G* = *C*, respectively. Therefore, division by the sequence length can serve as an indicator for the GC content. *n* = 2: mCGR with 50 % GC content in gray. For *n* = 4 and *n* = 6, the GC content of 50% is shown in green.

First, we consider the simple case for 50 % GC content. Depending on the order of the nucleotides at the edges of the mCGR, two patterns can evolve, as shown in Figure 4A. A GC content of 50% is shown in gray, for a word length of *n* = 2 and *n* = 4. If *A* and *T* are the opposite of each other, the resulting patterns are bars. While if they are positioned in diagonal directions, we get higher-order, more complex, flower-like patterns. The emerging patterns can be explained by the most simple case, namely the bars. We can reduce the mCGR to the first row and assign *A* = *T* = 0 and *G* = *C* = 1. We then convert the characters of a string to their binary representations. By using this simple encoding, new patterns of alternating ones and zeros emerge. For the first character in the sequence, we get an alternating pattern of zeroes and ones, while for the second character, we end up with alternating two zeros and two ones. Similar patterns occur for the next characters (see Figure 4 B. To retrieve the GC content, we calculate the sum of a column and divide it by the sequence length. By using this simple algorithm, we cannot only retrieve sequences with exactly 50% GC content but also defined intervals of interest. For the calculation of these patterns, we use the generator matrix with *A* = *T* = 0 and *C* = *G* = 1 to $\overset{0}{1}\ \overset{0}{1}$. However, we can also make use of our mathematical model in Equation (8c), which is not used for a complete motif but for the occurrence of a single character. For increasing word length, we have to double the size of the original matrix and either append or prepend the generator matrix (a single character respectively), as shown in Equation (3).

(3) $$\begin{eqnarray*} D ^{n} &=& {1\!\!1}^{2^{n-1}} \otimes D ^{1}+ D ^{n-1} \otimes {1\!\!1}^{2^1}\nonumber\\ &=& {1\!\!1}^{2^1} \otimes D ^{n-1}+ D ^{1} \otimes {1\!\!1}^{2^{n-1}} \nonumber\\ with\ D ^1 &=& \left\lbrace \begin{array}{lll}{\begin{bmatrix}0 & \quad 1 \\ 1 & \quad 0 \end{bmatrix}} & for &{\begin{bmatrix}A & \quad C \\ G & \quad T \end{bmatrix}} \\ \\ {\begin{bmatrix}0 & \quad 0\\ 1 & \quad 1 \end{bmatrix}} & for &{\begin{bmatrix}A & \quad T \\ G & \quad C \end{bmatrix}} \end{array} \right. \end{eqnarray*}$$

### Hamming distance

While the aforementioned constraints are very important for DNA storage systems, another constraint is of the utmost importance when we consider the code words for DNA computing, namely the Hamming distance. The Hamming distance between two sequences of equal length is the number of positions at which the corresponding symbols are different (49). In order to use code words for DNA computing or error correction, the Hamming distance of code words should be maximized. Using mCGR, it is also possible to integrate the Hamming distance as a constraint. To this end, we again make use of the initial matrix with the four nucleotides A, T, G, C. To calculate the Hamming distance for a single character, e.g. A, to all other nucleotides, we can set *A* = 0 and *G* = *T* = *C* = 1. To get the Hamming distance for a complete sequence, we have to append the corresponding Hamming distance for each nucleotide on each position (see equation (4)). Based on this, we can create a matrix with the Hamming distance of one single code word to all other code words.

(4) $$\begin{eqnarray*} H(s) ^n &=& \left\lbrace \begin{array}{@{}l@{\quad }l@{}}H (s)^1= B^1 & n = 1\nonumber\\ {1\!\!1}^{2^1} \otimes H(s)^{n-1} + B^1 \otimes {1\!\!1}^{2^{n-1}} & \text{otherwise} \end{array}\right.\\ with\ B^1 &=& \left\lbrace \begin{array}{lll} {\begin{bmatrix}0 & \quad 1 \\ 1 & \quad 1 \end{bmatrix}} & for & \quad A \\ \\ {\begin{bmatrix}1 & \quad 0 \\ 1 & \quad 1 \end{bmatrix}} & for & \quad T \\ \\ {\begin{bmatrix}1 & \quad 1 \\ 0 & \quad 1 \end{bmatrix}} & for & \quad G \\ \\ {\begin{bmatrix}1 & \quad 1 \\ 1 & \quad 0 \end{bmatrix}} & for & \quad C \\ \end{array} \right.. \end{eqnarray*}$$

To generate the complete table of the Hamming distance for all code words, we can apply Equation 3) for the GC content. To calculate the GC content, we used a binary representation of the nucleotides, where the GC content correspondents to the number of characters being A or T to the number of characters being G or C, which is equal to the Hamming distance for binary code words. This property enables the exchange of the generator matrix with a generator matrix $D_{2^1}$ for the Hamming distance of all nucleotides:

(5) $$\begin{eqnarray*} with\ D^1 = \begin{array}{lll} {\begin{bmatrix}0 & \quad 1 \quad & \quad 1 & \quad 1 \\ 1 & \quad 0 & \quad 1 & \quad 1\\ 1 & \quad 1 & \quad 0 & \quad 1\\ 1 & \quad 1 & \quad 1 & \quad 0 \end{bmatrix}} &for & rows=columns \\ \end{array} \end{eqnarray*}$$

### Implementation

We implemented all algorithms in R and run-time optimized versions for GC content and homopolymer/motif constraints in Java v. 12.0.1.

Since storing complete matrices would require a large amount of storage space, we decided to exclusively store the positions of the matrix elements affected by the constraints. It is, therefore, a lightweight form of a sparse matrix, where we do not assign values for the matrix elements. The set containing constrained words can be used to deduce the set of allowed words. We split equation (2) into two algorithms for tiling and doubling of the matrix. For the GC content, we implemented the approach shown in Figure 4B. The pseudocode for the algorithms can be found in Supplementary Material S9.

Our implementation allows any combinations of constraints for GC content (as a fixed number or interval), length of homopolymers, and undesired motifs (the latter provided in a FASTA formatted file). We also implemented a back-calculation algorithm for mCGR from elements in the matrix to sequences so that the allowed code words can be selected and stored as a FASTA file.

It is also possible to plot the results using the library JFreeChart v. 1.0.19 with JCommon v. 1.0.23. We implemented the CGR algorithm in Java, as a union square (height and width from −1 to 1). The coordinates of the CGR are stored as big integer fractions to increase the precision, and to avoid floating-point errors, by using the library Apache Commons Math v. 3.6.1.

We further implemented an option to generate codebooks that exclusively contain code words that can be concatenated without building forbidden motifs or homopolymers. Based on the approach proposed by Wang *et al.* (29), we excluded code words, which can build an undesired motif or homopolymer. To do this, we identified the words ending with the start of a motif or homopolymer matching at least half the length of the motif/homopolymer and the sequences beginning with the end of motif/homopolymer at least half the length. For instance, for the homopolymer *AAA* we removed all words ending with *AA* and starting with *AA*. Therefore, the matrix structure of the mCGR allowed us a quick identification of the words that should be excluded. Finally, the generated codebook can be used as a basis for a code. As an example, we implemented a lexicographic encoding/decoding based on our codebook, as proposed by Wang *et al.* (29) in python 3.

To encode an arbitrary input file using the lexicographic approach, the codebook is first sorted lexicographically, followed by partitioning the bits of the input file in blocks according to the information rate of the codebook. The decimal representation of a block is used as the index of the codebook for the mapping of binary sequences to code words. The decoding uses the same principle with a hashmap of code words as keys and the indices of the lexicographically sorted codebook as values.

### Comparison

First, we determined the coding rates of the codebooks generated by mCGR and lexicographical mCGR, based on the following equation (with *C* number of code words and *n* length of code words):

(6) $$\begin{equation*} code rate=\frac{log_2(|C|)}{n} \end{equation*}$$

We used different constraints and two sets of motifs, named scenario 1 and 2. While the first set contains 10 motifs with a length of 6, in scenario 2, we considered the motifs listed in MESA (19) with a maximum sequence length of 10 (overall 35 motifs).

To test and benchmark the mCGR-lexicographic code, we produced a benchmark dataset consisting of seven images in jpg format with different file sizes ranging from 300kB to 1.7MB. We used mCGR images generated with our R implementation to create the dataset (both the R script and the dataset are available under github.com/HFLoechel/ConstrainedKaos).

Based on this benchmark dataset, we encoded/decoded the benchmark dataset ten times and measured the runtime with the python package timit on a laptop (AMD Ryzen 5 3500 U, 16GB RAM) on a single CPU process. For comparison, we used the same benchmark dataset for encoding/decoding with the DNA Fountain code. For the mCGR approach, we first generated the codebooks in a word length of 10. Then we en- and decoded the seven benchmark files in different sizes, with the lexicographic code. For the DNA Fountain encoding/decoding, we first determined the minimum amount of packages needed for decoding for each file and applied the same restrictions without considering motifs. We measured the runtime 10 times for both codes. We disabled the Reed-Solomon for the DNA Fountain to make a fair comparison. For DNA Fountain, we noticed that with the minimal number of packages, the decoding did not work, and more packages are needed. To address this, we determined the minimal package number for decoding before the actual benchmark. Moreover, we used the encoded files and calculated the coding rates for each benchmark file, by dividing the number of nucleotides in each encoded file by the file size in bits. To make a fair comparison, we removed the header in the DNA Fountain codes. While DNA Fountain codes can not adhere to motif constraints, we analyzed the number of sequences in each encoded file affected by our motif selection in the two different scenarios.

## RESULTS

With our approach, it is possible to automatically generate all possible code words in a given length that take into account the GC content, the homopolymers, and the undesired DNA motifs.

### Mathematical model

In the following section, we summarize the mathematical background and equations that are used in our model.

For the alphabet Σ = {A,G,T,C}, the possible words of a length of *n* are the four possible nucleotides. For our mCGR approach, we have to split the concatenated string into substrings. For the simple case of all possible words in the length of two Σ^2^ and the initial mCGR for Σ^1^$\overset{A}{G}\ \overset{T}{C}$, we can get the first letter of all possible two-letter strings with the Kronecker product with the square matrix of ones $\overset{1}{1}\overset{1}{1}$= ${1\!\!1}^2$, which can be considered as an extension with any possible character:

(7) $$\begin{eqnarray*} P^2 &=& {1\!\!1}^{2^1} \otimes mCGR^1\nonumber\\ && ={\begin{bmatrix}1 & 1 \\ 1 & 1 \end{bmatrix}} \otimes {\begin{bmatrix}A & T \\ G & C \end{bmatrix}}\nonumber\\ && ={\begin{bmatrix}A & \quad T & \quad A & \quad T\\ G & \quad C & \quad G & \quad C\\ A & \quad T & \quad A & \quad T\\ G & \quad C & \quad G & \quad C\\ \end{bmatrix}} \end{eqnarray*}$$

We can proceed with the end of the strings in equal manner by exchanging both matrices:

(8) $$\begin{eqnarray*} A^2 &=& mCGR^1 \otimes {1\!\!1}^{2^1}\nonumber\\ &&= {\begin{bmatrix}A & \quad T \\ G & \quad C \end{bmatrix}} \otimes {\begin{bmatrix}1 & 1 \\ 1 &1 \end{bmatrix}}\nonumber\\ &&={\begin{bmatrix}A & \quad A & \quad T & \quad T\\ A & \quad A & \quad T & \quad T\\ G & \quad G & \quad C & \quad C\\ G & \quad G & \quad C & \quad C\\ \end{bmatrix}} \end{eqnarray*}$$

Finally, the addition of both matrices, generates the complete matrix *mCGR*^2^ for Σ^2^.

(9) $$\begin{eqnarray*} mCGR^2 &=& P^2+A^2\nonumber\\ && = {\begin{bmatrix}AA & \quad TA & \quad AT & \quad TT \\ GA & \quad CA & \quad GT & \quad CT\\ AG & \quad TG & \quad AC & \quad TC\\ GG & \quad CG & \quad GC & \quad CC \end{bmatrix}} \end{eqnarray*}$$

With this result, we can proceed to calculate the next mCGR:

(10) $$\begin{eqnarray*} P^3 &=& {1\!\!1}^{2^1} \otimes mCGR^2\nonumber\\ && A^3 = mCGR^1 \otimes {1\!\!1}^{2^3}\nonumber\\ && mCGR^3 = P^3+A^3 \end{eqnarray*}$$

We can now formulate the general equation to describe an mCGR by appending a single character as:

(11) $$\begin{eqnarray*} P^{n+1}={1\!\!1}^{2^1} \otimes mCGR^{n} \end{eqnarray*}$$

(12) $$\begin{eqnarray*} A^{n+1}= mCGR^1 \otimes {1\!\!1}^{2^{n}} \end{eqnarray*}$$

(13) $$\begin{eqnarray*} mCGR^{n+1} =A^{n+1}+P^{n+1} \end{eqnarray*}$$

While this approach allows the construction of Σ^*n*^ by appending a single letter, the model has to be extended to adhere to the different constraints. To this end, the mCGR model is defined as follows:

The mCGR model is defined as

(14) $$\begin{equation*} mCGR^n= P^n + A^n \end{equation*}$$

Prepending in the mCGR model is defined as

(15) $$\begin{equation*} P^n = {1\!\!1}^{2^1}\otimes mCGR^{n-1} \end{equation*}$$

while appending is defined as

(16) $$\begin{eqnarray*} A^n &= \left\lbrace \begin{array}{ll}mCGR^{1} \otimes {1\!\!1}^{2^{n-1}} & \text{ for Hamming and GC} \\ \\ mCGR^{n-1} \otimes {1\!\!1}^{2^{1}} & \text{ for hp and motifs} \end{array} \right. \end{eqnarray*}$$

The *mCGR*^1^ for the Hamming distance is defined as

(17) $$\begin{eqnarray*} mCGR^1 &={\begin{bmatrix} 0& \quad 1& \quad 1& \quad 1\\ 1& \quad 0& \quad 1& \quad 1\\ 1& \quad 1& \quad 0& \quad 1\\ 1& \quad 1& \quad 1& \quad 0\end{bmatrix}} \end{eqnarray*}$$

For the *mCGR*^1^ notation $\left[\overset{A}{G}\ \overset{T}{C}\right]$ applies:

(18) $$\begin{eqnarray*} mCGR^1 &= \left\lbrace \begin{array}{ll}\begin{bmatrix}0& \quad 0\\ 1& \quad 1\end{bmatrix} \text{ GC}\\ \cr B^1(i) \text{ Hamming (one sequence)}\end{array} \right. \end{eqnarray*}$$

The Hamming distance for the nucleotide at position i is defined as

(19) $$\begin{eqnarray*} B^1(i) &=& \left\lbrace \begin{array}{lll}\begin{bmatrix}0 & \quad 1 \\ 1 & \quad 1 \end{bmatrix} &for &A\\ \\ \begin{bmatrix}1 & \quad 0 \\ 1 & \quad 1 \end{bmatrix} &for &T\\ \\ \begin{bmatrix}1 & \quad 1 \\ 0 & \quad 1 \end{bmatrix} &for &G\\ \\ \begin{bmatrix}1 & \quad 1 \\ 1 & \quad 0 \end{bmatrix} & for & C \end{array} \right.. \end{eqnarray*}$$

For motifs or homopolymers for *n* = *i* and *i* = length of motif/homopolymer

(20) $$\begin{eqnarray*} mCGR^i \text{ with elements}\ e_{r,q} \end{eqnarray*}$$

is *e*_*r*, *q*_ = 1 for constrained substring, otherwise 0.

### Implementation

**Figure 5.**
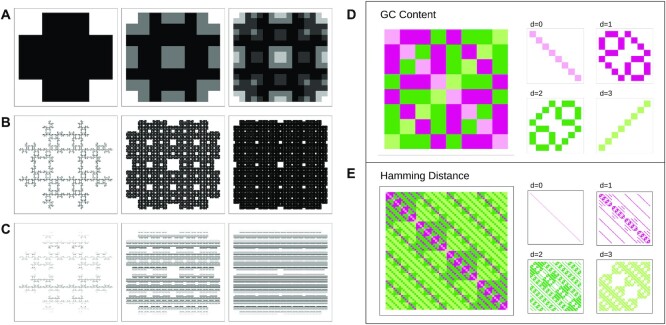
mCGR for different constraints: (A–C) mCGR of code words with different constraints. (**A**) Increasing word length for *hp* ≥ 2, 2 to 4 from left to right. A black area represents zero and a white area the highest number in the matrix. Thus, the code words with no homopolymers *hp* ≥ 2 are colored in black. (**B**) and (**C**) mCGR of *length* = 10, from left to right: constraints for *hp* ≥ 2, *hp* ≥ 3, and *hp* ≥ 4. White areas contain homopolymers. (**C**) Code words that does not fullfil 50 % GC content are excluded. (D and E) mCGR for the distance *d* with *length* = 3. (**D**) mCGR of GC content. (**E**) mCGR of Hamming distance. Left: complete representation. Right: different distances are shown.

We implemented all equations in R and Java. Figure 5A–C shows the mCGR of code words implementing different homopolymer constraints, with the usable sequences colored in black. Different fractal patterns emerge from the homopolymer constraints, which is caused by the fractal arrangement of the elements in the matrix (as described in Figure 2). A 50% GC content emerges as stripes in the representation, which is caused by the arrangement of the nucleotides.

Furthermore, we tested our implementation for several constraints concerning undesired motifs (see Supplementary Material S1 and S2). If a single nucleotide, for example, *A*, is forbidden, the Sierpinski triangle emerges. For other constraints, other fractals emerge, e.g. the T-square (a description of this fractal can be found in (50)).

Figure 5D and E shows the resulting representation for the GC content and Hamming distance for a word length of three.

A detailed view on the GC constraint as mCGR for different lengths and order of edges is given in Supplementary Material S3. The order of the edges has a high impact on the resulting pattern. To calculate the Hamming distance, we can apply the same equation for the GC content with a different generator matrix.

For the GC content, we found a similar pattern as for the Hamming distance of a binary code, as shown in (51). More precisely, when considering the following redefinition of *A* = *T* and *G* = *C*, one can notice that it is a transformation of the Hamming distance in a binary system. Hence, extracting all ones from the mCGR with diagonal order of *A* and *T* will result in the adjacency matrix for a hypercube. For other word lengths for the Hamming Distance see Supplementary Material S4. The results for the Hamming distance for a single word can be found in Supplementary Material S5.

To evaluate our approach, we generated a codebook containing 10 nucleotides (nt) long sequences without homopolymers longer than 2 nt, a GC content between 40–60 %, and a set of undesired motifs with relevance to sequence synthesis and sequencing. Due to the recursive definition of our model, a code word length of 10 nt covers all possible words shorter or equal to 10 nt as well as any longer code words. The resulting codebook contained 484 263 DNA sequences. We used the sequence evaluation tool MESA (19) to test whether the code words in the codebook fulfill the desired requirements. MESA is a web application for the assessment of synthetic DNA fragments with respect to homopolymers, GC content, *k*-mer repetitions, and undesired motifs. Using the MESA API, we evaluated the generated sequences concerning the fulfillment of the aforementioned constraints. MESA could not detect any unfulfilled constraints in our codebook. The input constraints and commands for the Java application as well as the MESA configuration are included in the GitHub repository. The MESA results confirmed that all sequences adhered to the restrictions.

### Comparison

In Supplementary Material S6, we compare our approach with other state-of-the-art methods for code word designs. In contrast to other approaches, our method is highly flexible in the number and type of constraints. This allows creating code words that adhere to a multitude of different constraints simultaneously. Furthermore, it is the only method so far that can generate code words adhering to undesired motifs. The GC content can be chosen as an interval or for a fixed percentage.

The Java implementation can be applied either to construct a codebook comprising all possible sequences in a given length under user-defined constraints, or all possible sequences which can freely be concatenated without forming undesired motifs or homopolymers, in order to use them for lexicographic encoding.

**Figure 6.**
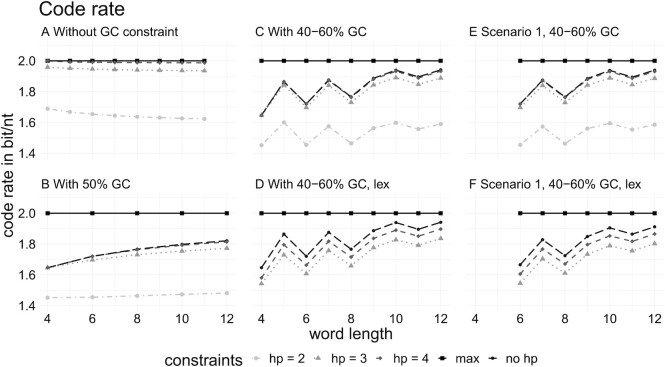
Code rate of mCGR with different constraints for different code word lengths.

Our approach allows the generation of codebooks with user-defined constraints. Therefore, we generated codebooks and calculated the corresponding code rates (Figure 6). For those, we varied the code word length, homopolymer length, and different GC content constraints with and without motif constraints. Additionally, we prepared codebooks for a lexicographic encoding (lex). These codebooks only contain code words that can freely be concatenated while still adhering to the constraints. In scenario 1 (Figure 6E and F), we added 10 undesired motifs with a length of 6. For choosing a GC interval of 40–60%, in different words (Figure 6C–F), a variation between odd and even numbers becomes visible, which is a result of the possible combinations to fit the interval. For example, code words of a length of 6 nucleotides, which have to adhere to an interval of 40–60% GC content, are always at exactly 50% GC to fit the criterion. Three nucleotides are either G or C, and the three remaining are either A or T. In contrast, an uneven code word length, e.g. 7, leaves more options. In this case, three or four nucleotides can be G or C and the remaining A or T to fit in the interval and vice versa. For a 10 nt code word, there are even more options to fit the criterion of a 40–60% GC content. Namely, there are three options: 4–6, 5–5 and 6–4. With an increase of the word length from 10 nt to 12 nt, the increase of the code rate is very small. Thus, regarding the runtime and memory, a code word length of 10 nt is reasonable for preparing a codebook based on the mCGR. The code rates for lexicographic encoding (lex) are shown in Figure 6D and F, which decreases as a result of a smaller codebook. In a second scenario (scenario 2), we created a lexicographic codebook with a set of 35 undesired motifs of a maximum sequence length of 10 nt (hp ≥ 4, GC 40–60%, a code word length of 10), leading to a code rate of 1.84. We used the three lexicographic codebooks (all with hp ≥ 4, GC 40–60%, code word length of 10), one with no motif constraint, one with the set of motifs of scenario 1, and one with the set of scenario 2, for encoding/decoding a benchmark dataset. The dataset consists of seven image files in different file sizes and compared them with the DNA Fountain code. In Supplementary Material S7, the results for the runtime benchmarks are presented. After preprocessing the codebooks, the lexicographic code has a linear runtime between a few seconds for encoding/decoding. The absolute encoding time is lower than for decoding. The encoding/decoding for DNA Fountain for our benchmark datasets was between minutes and it could be shown that the decoding has a non-linear runtime.

Additionally, we calculated the actual coding rate for each encoded file. For the DNA Fountain algorithm, we removed the 16 nt long headers for each package in advance to make the results comparable to the lexicographic encoding. The maximum code rate for DNA is 2 bits/nt while the lexicographic encoding has a constant code rate of 1.8 bits/nt in all three cases. Wang *et al.* (29) reached a code rate of 1.9 bits/nt with the same constraints. They dismissed all sequences ending with homopolymers of 3 nt and applied a concatenation scheme, avoiding the concatenation of sequences that are building homopolymers. Since we dismissed all code words that could form a motif or homopolymer, we can achieve a code rate of 1.88 for the same setting, while the implementation of our encoding/decoding decreased the actual code rate for our benchmark dataset to 1.8. The DNA Fountain code rate varies for the files with no visible pattern between 1.65 and 1.75 bits/nt. For the DNA Fountain encoded files, we counted the number of sequences affected with motifs for the two scenarios. For scenario 1, about 29% of the packages contained motifs in scenario 2, about 30%, independent of the file size. The relation of affected sequences is constant over the file size but varies with respect to the chosen scenarios. Detailed plots for the code rate comparison and the number of packages including motifs can be found in Supplementary Material S8.

## DISCUSSION

In the current study, we present a novel approach for generating codebooks that fulfill certain constraints that are important for DNA storage systems but have not been fully addressed by other approaches. These codebooks can be used as a basis for any DNA storage codes, e.g. error-correcting codes. The constraints include GC content (variable, strong and weak) as well as undesired motifs, which are particularly important for DNA syntheses and DNA sequencing. Our model calculates all possible code words in a given length, excluding those code words that do not adhere to the given constraints. Thus, for a given set of constraints, the maximal information density can be easily calculated.

The removal of undesired patterns is very complex. One can construct all possible code words in advance while discarding those with undesired motifs, homopolymers, or GC content. The remaining code words can further be used for encoding, as we did in our approach. In contrast, Fountain codes search for packages fulfilling all constraints during the encoding process. This step is very time-consuming as every code block needs to be checked against a number of motifs.

Our novel approach further offers the opportunity to calculate the Hamming distance, which is of high relevance for DNA computing (52). It is also possible to apply our approach for the Hamming distance for larger alphabets.

As a proof-of-concept, we used lexicographic encoding (29) to map binary strings to DNA code words. The matrix structure allows quick identification of sequences with a specific prefix or suffix. This enables a dynamic exclusion of matrix areas, depending on the last nucleotide(s) of the previously mapped code word. The mCGR-lexicographic code outperformed the DNA Fountain code, with respect to runtime and code rate. In our approach, we did not incorporate any error-correction due to the fact that the mCGR approach can be combined with any error-correction algorithm as mentioned before. Thus, we also disabled the error-correction in DNA Fountain to make a fair comparison. While Fountain-codes should be able to reach the theoretical maximum of 2 bits/nt, it turned out to be impossible to decode the data under this assumption. Additional packages for decoding need to be added to address this, which affected the code rate of the DNA Fountain code. In our lexicographical encoding, we discarded all sequences that can potentially form motifs/homopolymers, which decreased the code rate compared to the lexicographic approach of Wang *et al.* (29). Moreover, in two possible scenarios, on average, 30 % of the packages in the Fountain codes contain undesired motifs, which could lead to problems in synthesis or sequencing in the DNA storage systems.

To the best of our knowledge, this is the first algorithm that constructs code words that not only adhere to the commonly described constraints in the literature, but also to arbitrary undesired motifs, which play an important role in *in vitro* and *in vivo* studies of DNA codes and DNA storage systems, in particular for DNA synthesis and sequencing. As the structure of the matrix also allows the quick identification of reverse complementary code words, it can further be used in the design of DNA fragments for microarrays. The current JAVA implementation has some limitations regarding the code word and motif length. Without increasing the maximum allowed memory usage of JAVA (4GB for a system with 16GB main memory), a maximal code word length of around 12 nt can be calculated.

The mCGR method offers a new opportunity to construct code words and new perspectives regarding DNA codes. Besides the construction of specific code words, it can also be applied to visualize DNA-based encodings. The mCGR is a matrix representation of all possible code words for a given length Σ^*n*^ for DNA. Fractal patterns emerge from different constraints that can be mathematically described, and the back-calculation can be used to retrieve the code words.

## DATA AVAILABILITY

The implementation (for Java and R) is available at: http://mCGR.heiderlab.de. The source code is available at: https://github.com/HFLoechel/ConstrainedKaos.

## Supplementary Material

gkab1209_Supplemental_File
