# Supplementary material for: Fractal construction of constrained code words for DNA storage systems
Source: Nucleic Acids Res. 2021 Dec 15;50(5):e30. doi: 10.1093/nar/gkab1209 (PMC8934655; doi:10.1093/nar/gkab1209)
Supplement: gkab1209_Supplemental_File [file gkab1209_supplemental_file.pdf]

Fractal Construction of Constrained Code Words for DNA Storage Systems

Hannah F. Löchel, Marius Welzel, Georges Hattab,
Anne-Christin Hauschild and Dominik Heider

April 2020

Abstract

This section contains illustrations of the different constraints and the resulting mCGR representations for homopolymers, motifs (Figure S1 and S2), GC content (Figure S3), as well as for the Hamming distance (Figure S4). The mCGR representations for the Hamming distance for single code words can be found in Figure S5. A comparison of DNA Fountain and mCGR-lexicographic is provided in Figure S7 and Figure S8 Section S9 contains the pseudocode of the algorithms.

S1 Motifs

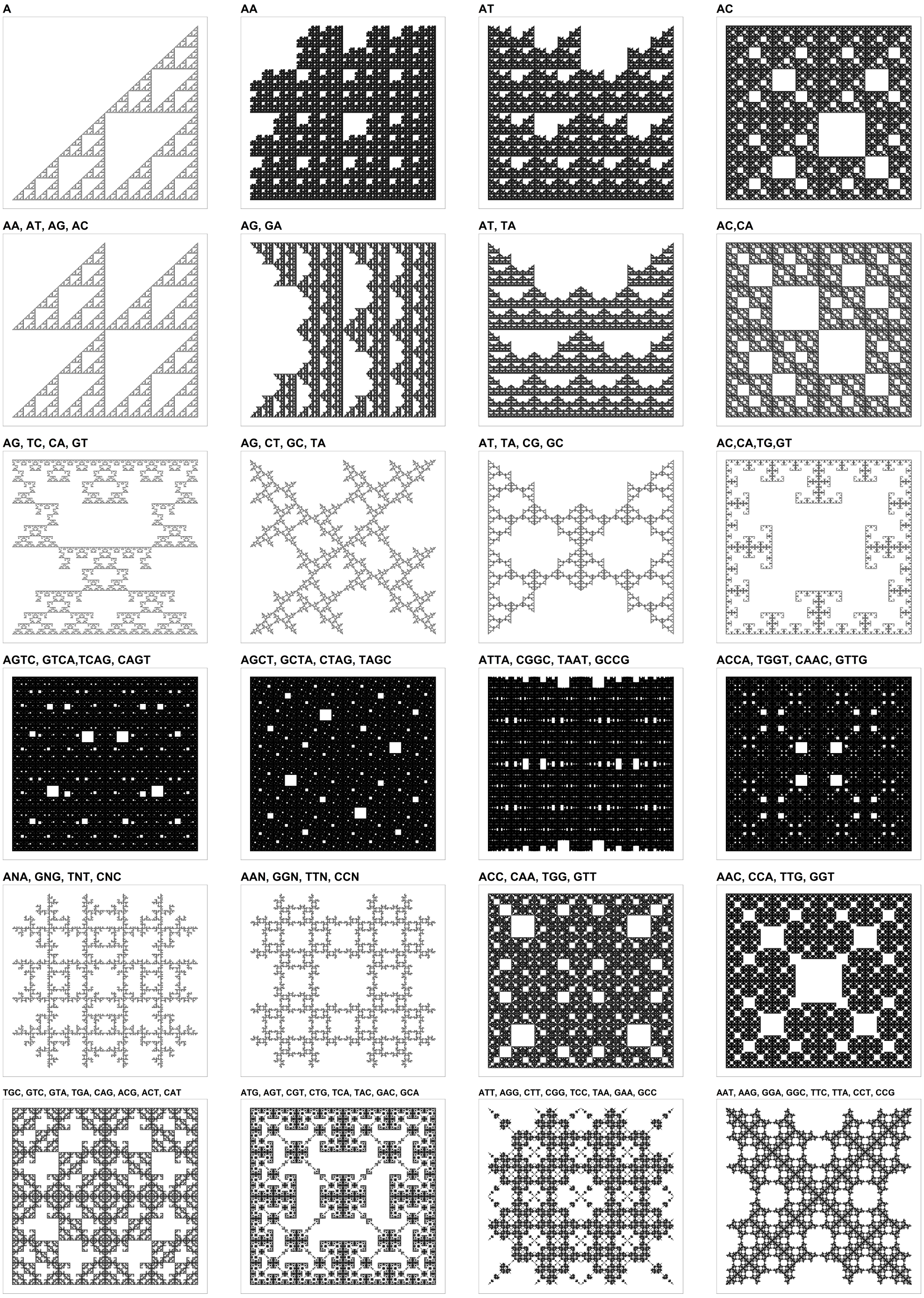

Figure S1: Examples for the mCGR representation of code words concerning motifs and homopolymers based on equation (algorithms 1 and 2):
$mCGR^n = \mathbb{1}^{2^1} \otimes mCGR^{n-1} + mCGR^{n-1} \otimes \mathbb{1}^{2^1}$. The code words fulfilling the constraints are shown in black.

S2 Amino Acids

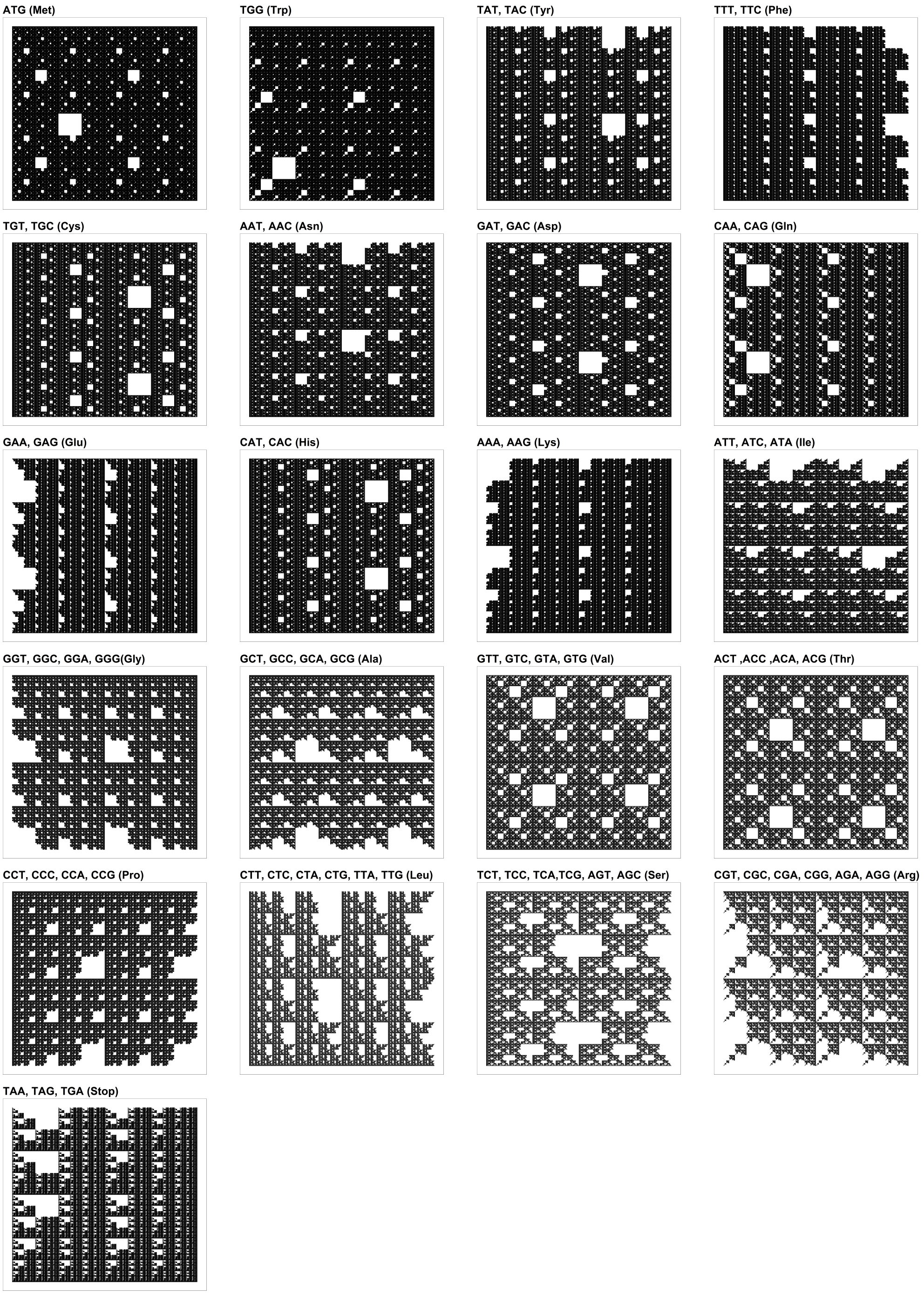

Figure S2: Amino acid codons based on equation (algorithms 1 and 2): $mCGR^n = \mathbb{1}^{2^1} \otimes mCGR^{n-1} + mCGR^{n-1} \otimes \mathbb{1}^{2^1}$. The code words fulfilling the constraints are shown in black.

S3 GC Content

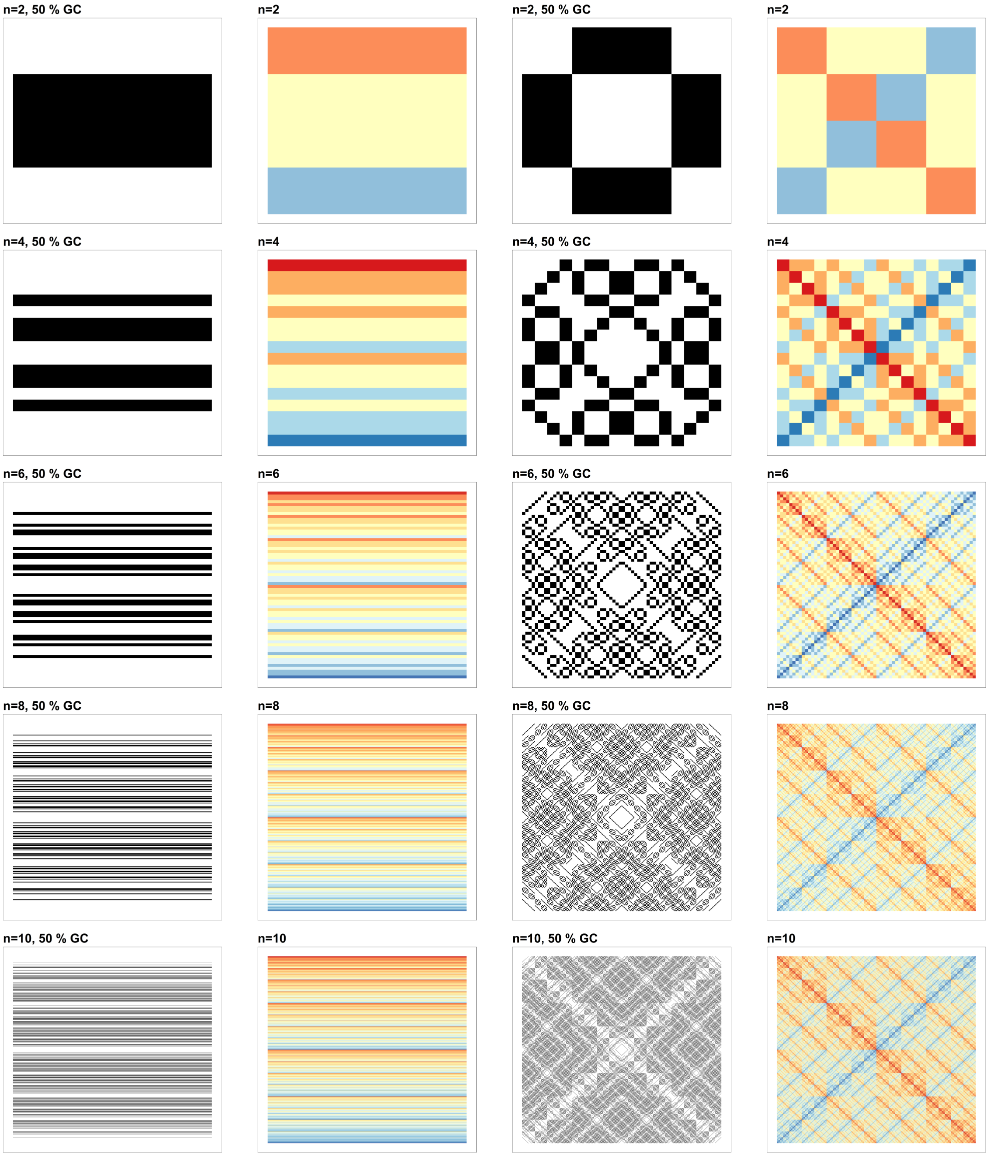

Figure S3: GC content for different word lengths (n) and arrangement of edges based on equation: $D^n = \mathbb{1}^{2^{n-1}} \otimes D^1 + D^{n-1} \otimes \mathbb{1}^{2^1}$
From top to bottom $n = 2, 4, 6, 8, 10$. 50 % GC content is shown in black. Left: edges arranged $\begin{smallmatrix} A & T \\ G & C \end{smallmatrix}$. Right: edges arranged $\begin{smallmatrix} A & C \\ G & T \end{smallmatrix}$. Based on the generator matrix $A = T = 0$ and $C = G = 1$. For the first generator matrix algorithm 3 can be applied.

S4 Hamming Distance

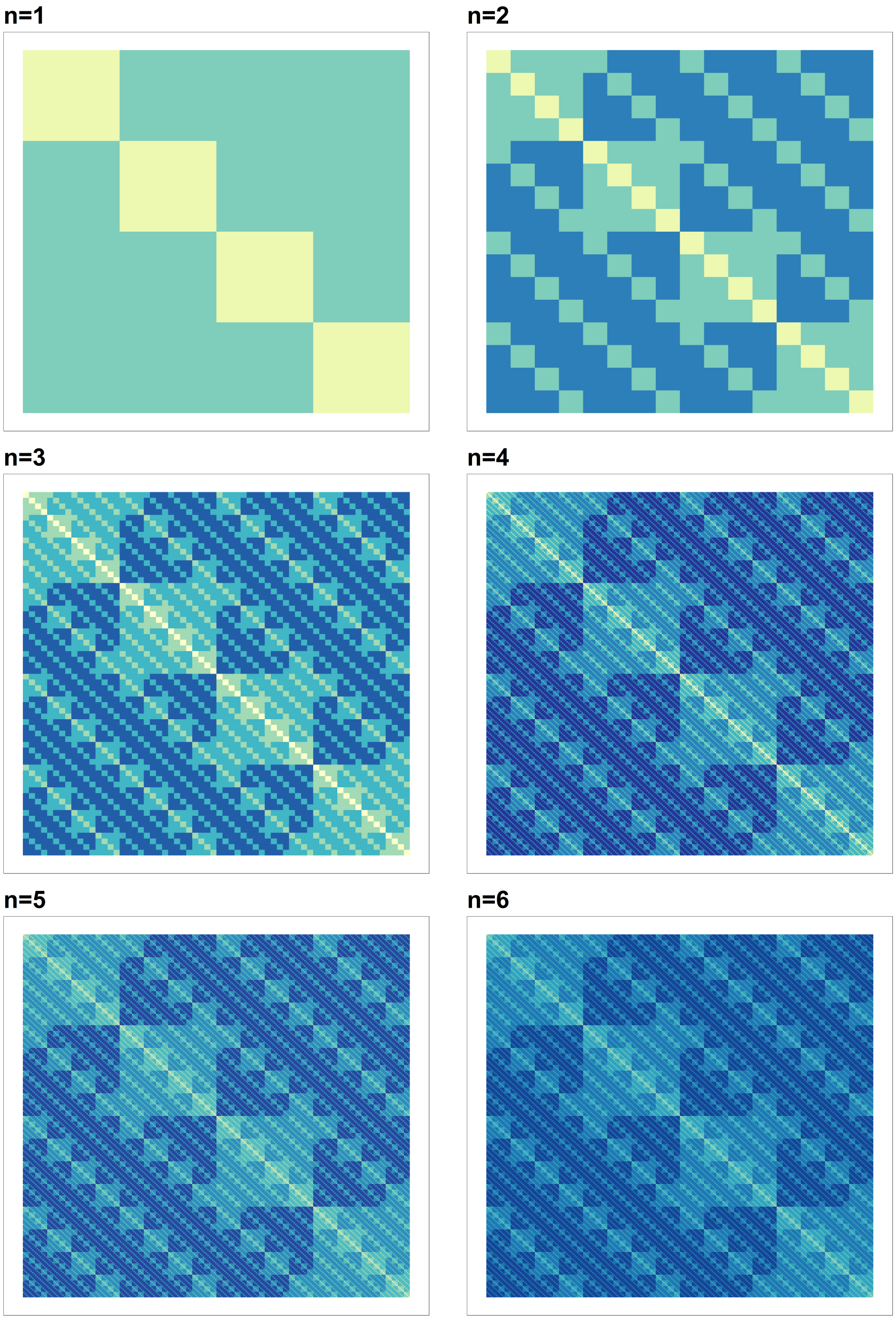

Figure S4: Hamming distance for different word length (n) based on equation: $D^n = \mathbb{1}^{2^{n-1}} \otimes D^1 + D^{n-1} \otimes \mathbb{1}^{2^1}$ and the generator matrix $\begin{smallmatrix} 0 & 1 & 1 & 1 \\ 1 & 0 & 1 & 1 \\ 1 & 1 & 0 & 1 \\ 1 & 1 & 1 & 0 \end{smallmatrix}$.

S5 Hamming Distance for Single Sequences

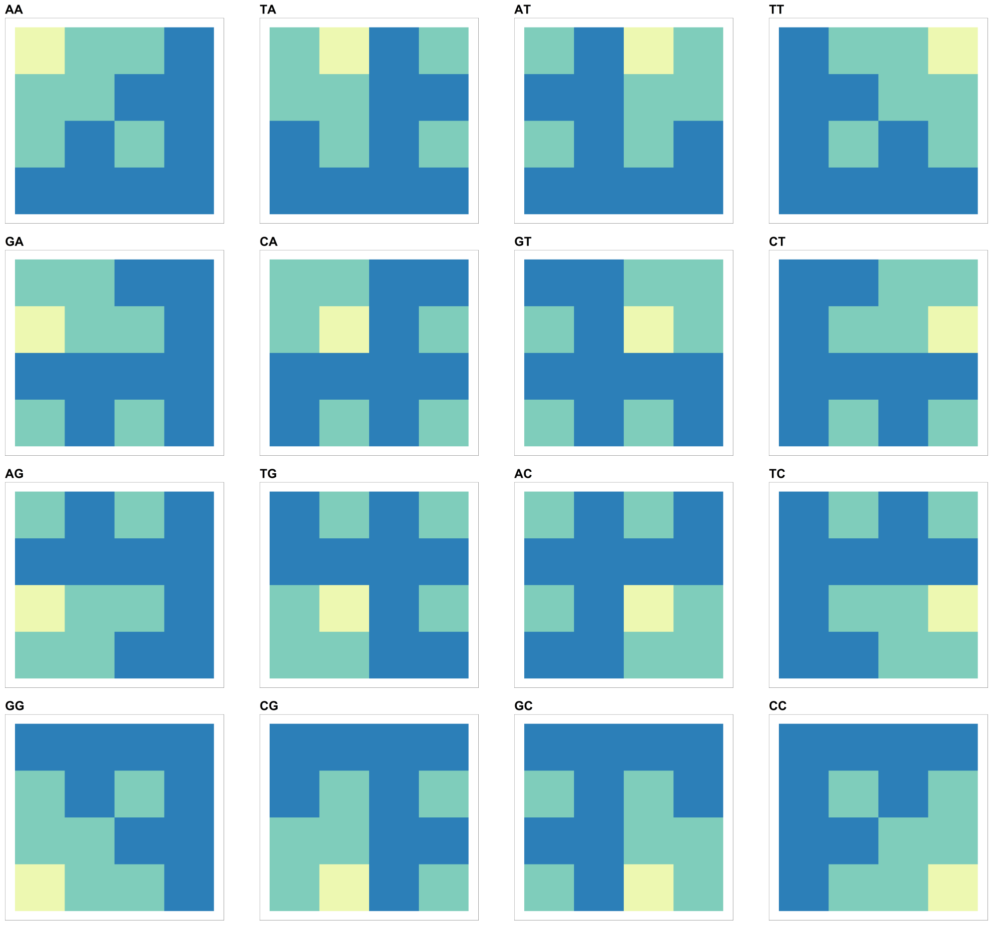

Figure S5: Hamming distance for single sequences. The matrix arrangement corresponds to the mCGR arrangement based on equation:
$H(s)^n = \mathbb{1}^{2^1} \otimes H(s)^{n-1} + B^1 \otimes \mathbb{1}^{2^{n-1}}$.

S6 Comparison of DNA Code Word Algorithms

Table 1: Comparison of DNA code word algorithms

	GC content	Homopolymers	Hamming	Undesired motifs
**Heuristic**				
Limbachiya et al. [1]	Strongly, Variable	>1	Variable	-
Gaborit and King [2]	Strongly, Variable	-	Variable	-
Wang et al. [3]	Weakly, Variable	Variable	Variable	-
Chee and Ling [4]	Strongly, Variable	-	Variable	-
**Deterministic**				
Song et al. [5]	Weakly, Fixed	>3	-	-
Immink and Cai [6]	Weakly, Fixed	Variable	-	-
Wang et al. [7]	Weakly, Fixed	>3	-	-
Dubé et al. [8]	Weakly, Fixed	>3	-	-
Our mCGR approach	Strongly and Weakly, Variable	Variable	Variable	Yes

S7 Benchmark

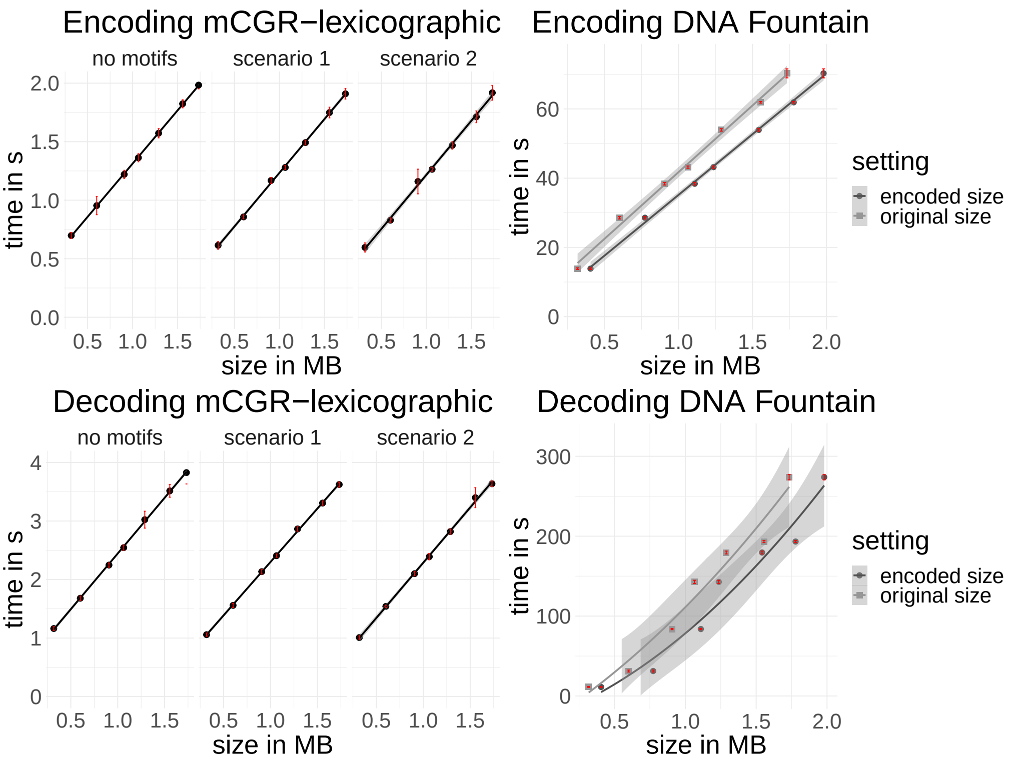

Figure S6: Benchmark of mCGR-lexicographic and DNA Fountain[9] for different file sizes. The dots with error bars represent the measurements, while the lines show a linear regression, except for the DNA Fountain decoding, in this case, the line represents a quadratic estimation. For DNA Fountain, the original file size and the encoded file size are plotted as for proper decoding more packages had to be generated.

S8 Comparison mCGR-lexicographic

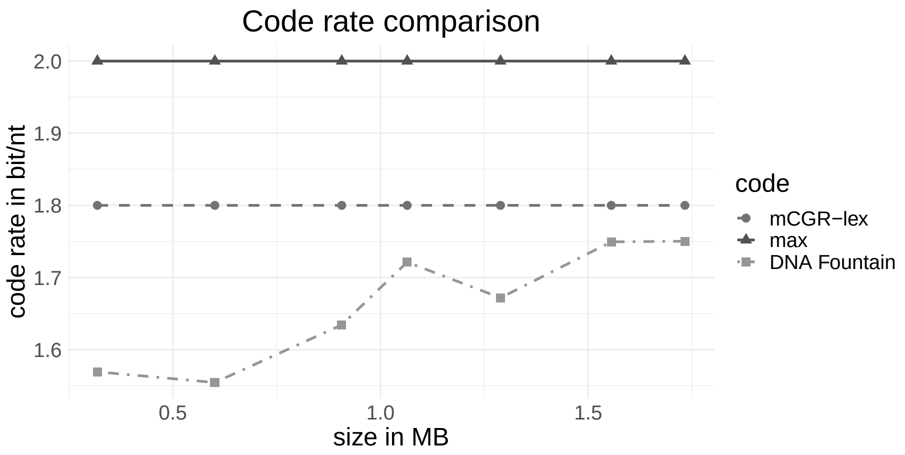

Figure S7: Comparison of code rate for DNA Fountain and mCGR-lexicographic.

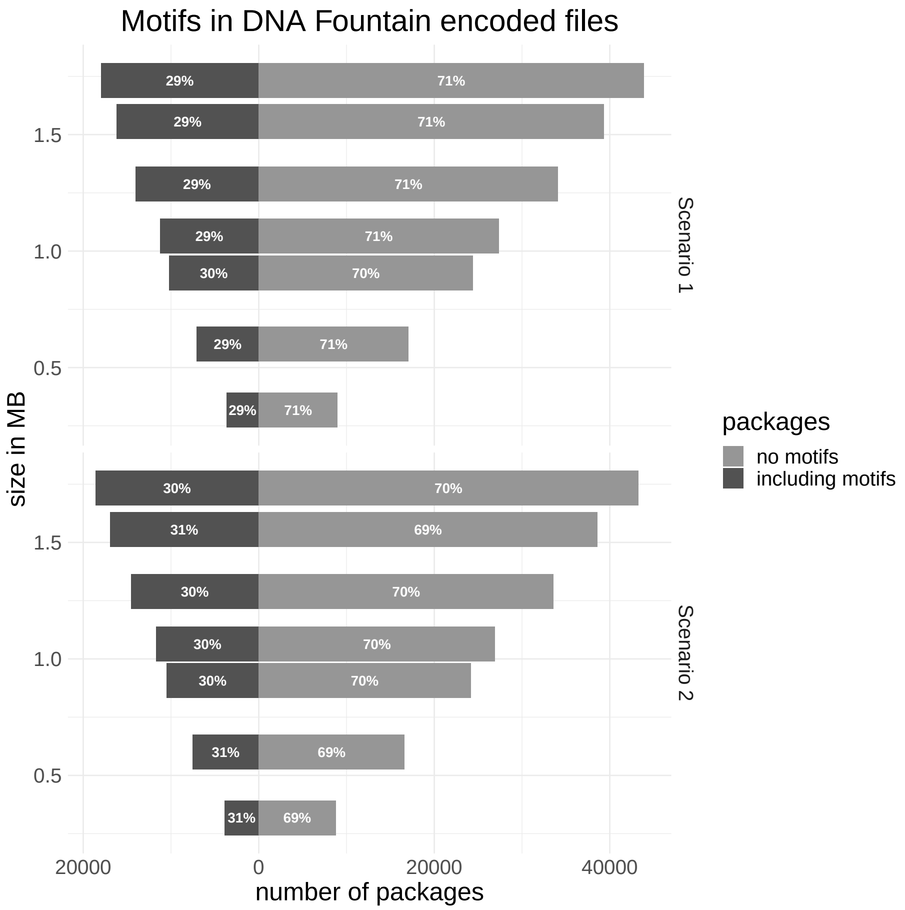

Figure S8: Ratio of packages containing motifs in DNA Fountain, for two possible scenarios.

S9 Algorithms

Algorithm 1 Tiling

```
function TILING(A,B)
    res ← dimension of B
    for i=0; i < res; i++ do
        for j = 0; j < res; j++ do
            if B[i][j]! = 0 then
                A[i][j] ← A[i][j] + B[i][j]
                A[i+res][j] ← A[i+res][j] + B[i][j]
                A[i][j+res] ← A[i][j+res] + B[i][j]
                A[i +res][j +res] ← A[i +res][j +res] + B[i][j]
    return A
```

Algorithm 2 Double sizing

```
function DOUBLESIZING(A)
    res ← dimension of A
    B ← [res*2][res*2]
    for i=0; i < res; i++ do
        for j = 0; j < res; j++ do
            if A[i][j]! = 0 then
                B[i * 2][j * 2] ← A[i][j]
                B[i * 2 +1][j * 2] ← A[i][j]
                B[i * 2][j * 2 +1] ← A[i][j]
                B[i * 2 +1][j * 2 +1] ← A[i][j]
    return B
```

Algorithm 3 Calculate GC content

```
function CALCULATEGCCONTENT(length, proportion)
    size ← power(2,length-1)
    content ← [size]
    for i=0; i < length; i++ do
        switcher ← 1
        position ← power(2,i)
        for j = 0; j < size; j++ do
            if jposition == 0 then
            content[j] = content[j] + switcher
    return content
```

References

[1] Dixita Limbachiya, Manish K Gupta, and Vaneet Aggarwal. Family of constrained codes for archival dna data storage. *IEEE Communications Letters*, 22(10):1972–1975, 2018.

[2] Philippe Gaborit and Oliver D King. Linear constructions for dna codes. *Theoretical Computer Science*, 334(1-3):99–113, 2005.

[3] Yanfeng Wang, Yongpeng Shen, Xuncai Zhang, and Guangzhao Cui. DNA codewords design using the improved NSGA-II algorithms. In *2009 Fourth International on Conference on Bio-Inspired Computing*. IEEE, oct 2009. doi: 10.1109/bicta.2009.5338158.

[4] Yeow Meng Chee and San Ling. Improved lower bounds for constant gc-content dna codes. *IEEE Transactions on Information Theory*, 54(1):391–394, 2008.

[5] Wentu Song, Kui Cai, Mu Zhang, and Chau Yuen. Codes with run-length and gc-content constraints for dna-based data storage. *IEEE Communications Letters*, 22(10):2004–2007, 2018.

[6] Kees A Schouhamer Immink and Kui Cai. Efficient balanced and maximum homopolymer-run restricted block codes for dna-based data storage. *IEEE Communications Letters*, 23(10):1676–1679, 2019.

[7] Yixin Wang, Md. Noor-A-Rahim, Erry Gunawan, Yong Liang Guan, and Chueh Loo Poh. Construction of bio-constrained code for DNA data storage. *IEEE Communications Letters*, 23(6):963–966, jun 2019. doi: 10.1109/lcomm.2019.2912572.

[8] Danny Dubé, Wentu Song, and Kui Cai. Dna codes with run-length limitation and knuth-like balancing of the gc contents. In *Symposium on Information Theory and its Applications (SITA), Japan*, 2019.

[9] Yaniv Erlich and Dina Zielinski. Dna fountain enables a robust and efficient storage architecture. *Science*, 355(6328):950–954, 2017.
